# Antimicrobial susceptibility profiles of *Staphylococcus* spp. contaminating raw goat milk

**DOI:** 10.14202/vetworld.2021.1074-1079

**Published:** 2021-05-05

**Authors:** Abimael E. Silva Júnior, Priscylla C. Vasconcelos, Mauro M. S. Saraiva, Lauro Santos Filho, Núbia M. V. Silva, Patricia E. N. Givisiez, Celso J. B. Oliveira

**Affiliations:** 1 Department of Animal Science, College for Agricultural Sciences, Federal University of Paraiba, Brazil; 2 Department of Pharmaceutical Sciences, College of Health Sciences, Federal University of Paraiba, Brazil

**Keywords:** antimicrobial resistance, dairy goats, food safety, *Staphylococcus*

## Abstract

**Background and Aim:**

Antimicrobial resistance poses a major threat to global public health. Foodstuff of animal origin can serve as potential vehicles for the dissemination of antimicrobial-resistant bacteria and resistance genes to consumers. In view of the lack of knowledge about antimicrobial resistance in bacteria associated with goat milk, the aim of this study was to report species-level identification and antimicrobial susceptibility profiles of a large collection of *Staphylococcus* spp. isolates recovered from raw goat milk in Brazil.

**Materials and Methods:**

A total of 434 *Staphylococcus* spp. isolates originated from 510 goat milk samples in Northeast Brazil were investigated. The isolates were obtained by conventional microbiological methods. Species identification and antimicrobial susceptibility testing were performed by means of a semi-automated system using a panel for biochemical tests and broth microdilution method for 19 antimicrobial drugs.

**Results:**

Although *Staphylococcus aureus* (22.6%) accounted for the majority of the isolates, a total of 13 different non-*aureus* staphylococci spp. were identified. High resistance rates against erythromycin (40.8%), and the beta-lactams ampicillin (45.9%) and penicillin (42.9%) were observed among *S. aureus* isolates. The most significant findings were related to the resistance against quinupristin-dalfopristin, a drug of last resort used in human medicine to treat infections caused by vancomycin-resistant *S. aureus* and enterococci.

**Conclusion:**

The high diversity of *Staphylococcus* spp. showing phenotypic resistance against different antimicrobial drugs encourages further investigations on the real impact of these bacteria as reservoirs of antimicrobial resistance genes to consumers. Furthermore, the potential impact of technological processes, such as pasteurization, fermentation, and maturation, on the maintenance and dissemination of antimicrobial resistance among the microbial populations in milk and dairy products must also be investigated.

## Introduction

Paraiba is the leading goat milk producer state in Brazil, with a total production of 5.63 million litters, representing 23.9% of the revenue attributed to the whole Brazilian goat milk sector [1]. In Paraiba State, milk production is based on small-scale extensive or semi-extensive farming in semiarid regions, in which dairy goats are raised on native Caatinga vegetation (seasonal dry forest) and fed cactus pear (*Opuntia ficus*) as fodder [2]. As part of government programs to improve the agricultural sector in low-income areas, goat raw milk is purchased from smallholders, pasteurized in small-scale dairy plants, and distributed to schools and day care units [3].

Although dairy goat production plays an important socioeconomic role in low-income Northeast Brazil, very little is known regarding staphylococcal contamination in milk produced by smallholders in semi-arid Brazil. Besides, the fact that staphylococci are leading foodborne pathogens worldwide due to the capacity of some strains to produce toxins [4,5], including heat-stable enterotoxins that can resist the pasteurization process [6], antimicrobial-resistant *Staphylococcus* spp. are of public health concern. Although the extensive use of antimicrobials in human medicine is a major driver for the emergence and dissemination of antimicrobial resistance [7], foodstuffs might play a potential role in the dissemination of antimicrobial-resistant bacteria and resistance genes to human populations consuming these products [8]. The emergence and dissemination of antimicrobial resistance in food bacteria are favored by increased selection pressure caused by the extensive use of antimicrobial drugs in food animal production systems [9]. Subclinical intramammary infections caused by *Staphylococcus* spp., the most common disease affecting the dairy industry worldwide [10], are associated with continuous use of antibiotics in dairy cattle [11]. However, information on antimicrobial resistance levels in *Staphylococcus* associated with intramammary infections in goats is limited [12]. Another critical limitation is the lack of knowledge on the spp. of *Staphylococcus* associated with milk and dairy products, as the majority of the studies identify isolates as *Staphylococcus aureus* and non-*aureus* staphylococci (NAS) spp. The identification of the most frequent *Staphylococcus* spp. and the determination of their antimicrobial susceptibility rates are important for controlling and prevention purposes at both farm and industry levels [13].

Staphylococci are highly capable of acquiring resistance to several classes of drugs, including the highest priority critically important antimicrobials (HPCIA), which are drugs of last resort to treat serious bacterial infections in people[13]. Estimates of the economic impact of antimicrobial resistance to the global economy reach dreadful numbers. The World Bank announced that antimicrobial resistance could affect the global GDP by between 1.1 and 3.8% points by 2050 [14]. According to the World Economic Forum [15], the estimated costs for antimicrobial resistance can reach up to $100 trillion by 2050, and the number of deaths could exceed 300,000 people per year. Microbial contamination of animal-derived foods may occur directly through infected animals or may result from cross-contamination during processing, food transport, and storage [16,17]. Therefore, investigations on antimicrobial susceptibility patterns in food contaminating bacteria are important to monitor foodstuffs as potential vehicles for dissemination of antimicrobial resistance genes to consumers.

The aim of the present study was to investigate the most common spp. of *Staphylococcus* contaminating goat milk and to assess their susceptibility profiles against antimicrobials, including some drugs commonly used in human medicine to treat staphylococcal infections.

## Materials and Methods

### Ethical approval and Informed consent

Ethics approval was not required for this study. Verbal consent was obtained from farmers allowing milk samples to be collected during routine milking procedures.

### Study period and location

The milk samples were collected from Cariri region, Paraíba State, Northeastern Brazil from March 2015 to February 2016. Milk samples were processed at the Laboratory for Animal-derived Foods (LAPOA) of the College for Agricultural Sciences, Federal University of Paraíba (CCA/UFPB).

### Study design

*Staphylococcus* spp. isolates originated from goat milk (n=434) were investigated in this study. The isolates were recovered from 510 milk samples ­collected in Cariri, a semi-arid region in Paraiba State, Northeast Brazil. Samples (10 mL) were streaked onto Baird Parker agar (HiMedia, India) and incubated at 37°C for 24-48 h Colonies showing typical and non-typical *Staphylococcus* characteristics were tested for catalase, oxidase and examined microscopically after conventional Gram staining procedure. Confirmed *Staphylococcus* isolates were further identified by means of a biochemical panel (Combo PC33, Siemens Healthcare, Germany), including the following tests: Crystal violet reaction, nitrate reduction, novobiocin resistance, Voges–Proskauer, sensitivity to optochin, alkaline phosphatase, bile esculin, production of pyrrolidone, dehydrolyzation of arginine, breakage of arginine, glycosidase production, hydroxylation of indoxyl phosphatase, urea, phenol red, growth at 6.5% NaCl, bacitracin resistance, use of pyruvate, presence of β-lactamase, and hemolysis. Species identification was based on automatic interpretation of the biochemical test results by AutoScan4 software (Siemens Healthcare, Germany).

### Antimicrobial susceptibility test

Phenotypic antimicrobial susceptibility was assessed by means of a semi-automated microdilution method (Autoscan4, Siemens Healthcare) for the following drugs and their respective cutoff values (μg/mL): Ampicillin/sulbactam (Sam) (≥32/16); ampicillin (Amp) (≥0.5); amoxicillin/clavulanate de K (≥8/4); ceftriaxone (≥64); clindamycin (Cli) (≥4); ciprofloxacin (Cip) (≥4); daptomycin (Dap) (≥2); erythromycin (Eri) (≥8); gentamicin (Gen) (≥16); nitrofurantoin (≥128); quinupristin/dalfopristin (QD) (≥4); levofloxacin (Lvx) (≥8); linezolid (Lzd) (>4); moxifloxacin (Mxf) (≥8); oxacillin (Oxa) (≥0.5 para SCN e ≥4 para *S. aureus/Staphylococcus lugdunensis*); penicillin (Pen) (≥0.25); rifampin (Rif) (≥4); trimethoprim/sulfamethoxazole (Sxt) (≥4/76); and tetracycline (Tet) (≥16). The interpretation of the results was performed according to the Clinical and Laboratory Standards Institute criteria [18]. *S. aureus* ATCC 25923 was used as control.

Isolates showing susceptibility to all drugs were considered pan-susceptible and those resistant to three or more different antimicrobial classes were considered multidrug-resistant (MDR) isolates [19].

## Results

*S. aureus* (n=98; 22.6%) and *Staphylococcus epidermidis* (n=71; 16.4%) were the most frequent spp. cultured from raw goat milk (Table-1). A total of 13 different spp. of coagulase-negative staphylococci were identified in goat milk samples. The high frequency of *Staphylococcus hyicus* spp. (n=22; 5.1%) is noteworthy. Table-2 shows absolute and relative frequencies of antimicrobial susceptibility of *S. aureus* and NAS. Pan-susceptibility was observed in 155 (35.7%) isolates. Interestingly, we detected NAS showing resistance to the highest priority critically important antimicrobials that are used exclusively to treat skin and urinary tract infections in humans [20,21].

**Table-1:** Coagulase-positive *Staphylococcus* and coagulase-negative *Staphylococcus* spp. cultured from raw goat milk from Northeast Brazil.

*Staphylococcus* spp.	Number of isolates	%
*Staphylococcus aureus*	98	22.6
*Staphylococcus hyicus*	22	5.1
*Staphylococcus intermedius*	41	9.4
*Staphylococcus epidermidis*	71	16.4
*Staphylococcus simulans*	46	10.6
*Staphylococcus haemolyticus*	32	7.4
*Staphylococcus hominis* subsp*. hominis*	14	3.2
*Staphylococcus saprophyticus*	10	2.3
*Staphylococcus lugdunensis*	16	3.7
*Staphylococcus sciuri*	21	4.8
*Staphylococcus schleiferi* subsp. *schleiferi*	21	4.8
*Staphylococcus cohnii* subsp. *cohnii*	07	1.6
*Staphylococcus xylosus*	16	3.7
*Staphylococcus auricularis*	09	2.1
*Staphylococcus warneri*	09	2.1
*Staphylococcus cohnii* subsp. *urealyticum*	01	0.2
Total	434	100

**Table-2:** Antimicrobial susceptibility profiles of *Staphylococcus* *aureus* and non-*aureus* staphylococci isolated from goat milk in Paraiba State, Northeast Brazil.

Class	Antimicrobials	*Staphylococcus aureus* (%)	Non-*aureus* staphylococci (%)
Beta-lactams	Amp	45 (45.9)	123 (36.6)
	Pen	42 (42.9)	136 (40.5)
	Oxa	17 (17.4)	67 (19.9)
	Cro	7 (7.1)	17 (5.1)
Beta-lactam/beta-lactamase inhibitors	Amc	4 (4.1	21 (6.3)
	Sam	11 (11.2)	47 (14.0)
Sulfonamides	Sxt	3 (3.1)	25 (7.4)
Fluoroquinolones	Lvx	6 (6.1)	37 (11.0)
	Mxf	4 (4.1)	11 (3.3)
	Cip	8 (8.2)	21 (6.3)
Lipopeptides	Dap	16 (16.3)	63 (18.8)
Nitrofurantoin	Nft	1 (1.0)	23 (6.9)
Ansamycins	Rif	10 (10.2)	39 (11.6)
Streptogramins	QD	9 (9.2)	42 (12.5)
Lincosamides	Cli	20 (20.4)	99 (29.5)
Macrolides	Eri	40 (40.8)	115 (34.2)
Aminoglycosides	Gen	13 (13.3)	27 (8.0)
Oxazolidines	Lzd	3 (3.1)	36 (10.7)
Tetracyclines	Tet	17 (17.4)	87 (25.9)

Sam=Ampicillin/sulbactam; Amp=Ampicillin; Amc=Amoxicillin/clavulanate de K; Cro=Ceftriaxone; Clindamycin; Cip=Ciprofloxacin; Dap=Daptomycin; Eri=Erythromycin; Gen=Gentamicin; Nft=Nitrofurantoin; QD=Quinupristin/dalfopristin; Lvx=Levofloxacin; Lzd=Linezolid; Mxf=Moxifloxacin; Oxa=Oxacillin; Pen=Penicillin; Rif=Rifampin; Sxt=Trimethoprim/sulfamethoxazole; Tet=Tetracycline

In our study, 4.1% of *S. aureus* and 6.3% of NAS isolates were resistant to amoxicillin/clavulanate. We also detected resistance against QD among the NAS. This group showed higher resistance rates against Cli and Eri (p<0.05) than *S. aureus*.

Table-3 shows the frequency of MDR profiles among *Staphylococcus* spp. isolated from goat milk. A total of 134 (30.9%) MDR isolates were detected. One *S. aureus* isolate was resistant to 14 drugs (Sam, Amp, Dap, Lvx, Mxf, Rif, Syn, Cli, Cip, Eri, Gen, Lzd, Oxa, Pen, Sxt, and Tet).

**Table-3:** Multidrug-resistant *Staphylococcus aureus* and non-*aureus* staphylococci spp. isolated from goat milk in Paraiba State, Northeast Brazil.

Number of drug classes	MDR *Staphylococcus aureus* (%)	MDR non-*aureus* *staphylococci* (%)
3	5 (5.1)	32 (9.5)
4	8 (8.2)	11 (3.3)
5	0 (0.0)	9 (2.7)
6	2 (2.0)	10 (2.9)
7	5 (5.1)	18 (5.3)
8	5 (5.1)	10 (2.9)
9	0 (0.0)	7 (2.0)
10	1 (1.0)	4 (1.2)
11	1 (1.0)	5 (1.5)
12	0 (0.0)	1 (0.3)
Total	27.5	31.6

MDR=Multidrug-resistant

## Discussion

The high occurrence of *S. aureus* in our study was expected as this species is commonly isolated from dairy animals worldwide. Nonetheless, this finding is of public health significance, as *S. aureus* is among the main *Staphylococcus* species causing food poisoning in humans due to the production of heat-stable enterotoxins [6]. The observed high frequency of *S. hyicus* among the goat milk samples in the present study is surprising, since *S. hyicus* has not been commonly detected in milk. Considering that *S. hyicus* originates from skin sources [22], the high occurrence of this spp. in milk could be related to hygiene deficiencies in teat cleaning and disinfection during milking practices [23] that can also increase overall milk contamination. This is corroborated by the fact that some species commonly associated with skin infections in both animals and humans [24] were detected in our study, such as *Staphylococcus xylosus*, *Staphylococcus schleiferi*, and *Staphylococcus simulans*, and have been reported as emerging zoonotic pathogens [25-27].

*S. aureus, S. epidermidis*, an*d S. hyicu*s are leading pathogenic staphylococci in veterinary medicine [28]. In dairy goats, *S. epidermidis, S. simulans*, and *S. xylosus* have been commonly isolated from intramammary infections [12,29,30]. The high frequency of *S. epidermidis* in our study is in accordance with a recent study in Mexico [31] reporting this organism as the main *Staphylococcus* spp. cultured from goat milk. *S*. *epidermidis* high frequency in goat milk has also been reported in previous studies, possibly associated with subclinical mastitis in dairy goats [29].

According to a previous investigation on the antimicrobial susceptibility of a large set of *S. aureus* from foodstuffs (1150 samples), the highest resistance rates were observed against Pen (83%), Lzd (67.7%), and Eri (52.1%); however, significant differences were observed according to the type of food [32].

The detection of *S. aureus* and NAS resistance to amoxicillin/clavulanate suggests that some *Staphylococcus* strains found in raw goat milk can harbor different resistance mechanisms, such as serine-β-lactamase production, including extended-spectrum β-lactamases that could hydrolyze later-generation cephalosporins and carbapenemases. Thus, NAS can be considered reservoirs of antimicrobial resistance, although *S. aureus* has been considered the key spp. involved in both food poisoning outbreaks and clinical infections in humans. This is also corroborated by the high resistance rates observed in NAS isolated from food animal sources, particularly resistance against macrolides, Tet, and streptogramins [33].

Considering that quinupristin is a drug of last resort used to treat infections caused by vancomycin-resistant *S. aureus* and enterococci, and these are among the most rapidly growing multidrug-resistant bacteria affecting humans [34], further investigations on the quinupristin resistance mechanisms, these isolates are warranted.

Tet has been commonly used for therapeutic purposes on farm animals for decades and high rates of resistance against this drug have been observed among different organisms, including pathogenic bacteria [35].

The real impact of antimicrobial-resistant *Staphylococcus* spp. contaminating goat milk on the putative dissemination of antimicrobial resistance to humans is beyond the scope of this study. However, considering that pasteurization is a mild heat treatment, and the fact that milk is a suitable medium for the maintenance and growth of bacteria, it is plausible to consider that milk can serve as source of antimicrobial resistance genes to the human gut microbiota, supporting previous assumptions [36,37]. The ­rationale for this is based on the many mechanisms of horizontal transference of antimicrobial resistance genes among bacteria, such as plasmids, transposons, and integrons.

Finally, staphylococci can serve as reservoirs of antimicrobial resistance genes to milk microorganisms. A large number of antimicrobial resistance genes have been identified in lactic acid bacteria [38,39]. Increased rates of antimicrobial resistance in lactobacilli and related bacteria can pose a risk to consumers as they can disseminate resistance genes to pathogenic bacteria.

## Conclusion

The high antimicrobial resistance rates in *S. aureus* and NAS in goat milk produced in the investigated region reveal the potential role of milk as a reservoir of antimicrobial resistance genes. The findings encourage further investigation of putative effects of technological processes, such as pasteurization, fermentation, and maturation, on the maintenance and dissemination of antimicrobial resistance genes in the microbiota of goat milk and dairy products. Such knowledge could be important to mitigate the spread of antimicrobial resistance in the dairy industry. Finally, our results highlight the importance of on-herd hygiene measures to reduce microbial contamination in milk produced by smallholders and the continuous awareness of producers toward antimicrobial stewardship.

## Authors’ Contributions

CJBO and LSF: Study conception and design. AESJ, MMSS, NMVS, and PCV Acquisition of data, analysis and interpretation of data. PENG, AESJ and LSF: Drafting of the manuscript: PENG, NMVS and CJBO: Supervision. PENG and CJBO: Critical revision. All authors read and approved the final manuscript.
